# Erythrodermic Psoriasis With Anasarca and Lymphadenopathy Mimicking Lymphoma

**DOI:** 10.7759/cureus.117556

**Published:** 2026-10-06

**Authors:** Noren Din

**Affiliations:** 1 Internal Medicine, MultiCare Allenmore Hospital, Tacoma, USA; 2 Internal Medicine, MultiCare Tacoma General Hospital, Tacoma, USA

**Keywords:** dermatopathic lymphadenopathy, erythrodermic psoriasis, generalized anasarca, lymphoma mimic, oral methotrexate

## Abstract

A 25-year-old man with chronic psoriasis presented with progressive erythroderma, anasarca with a reported 30-lb weight gain, and bulky lymphadenopathy, raising concern for lymphoma. Lymph node biopsy showed dermatopathic lymphadenopathy, and expert review of skin specimens favored an inflammatory process over cutaneous T-cell lymphoma. Low-dose methotrexate was started, followed by patient-reported improvement in function, edema, and skin changes. This case emphasizes recognizing severe inflammatory skin disease as a possible contributor to systemic illness and correlating the clinical presentation with pathology. Improvement after methotrexate supports clinical benefit but is not diagnostic of psoriasis.

## Introduction

The skin is the body's largest organ, and extensive disruption of its barrier function can lead to metabolic, hemodynamic, and thermoregulatory complications [1]. Erythrodermic psoriasis (EP) is a rare, severe variant affecting approximately 1-2.25% of patients with psoriasis [2]. Erythroderma may also be a manifestation of cutaneous T-cell lymphoma or systemic lymphoma, creating substantial diagnostic overlap with EP [3]. Lymphadenopathy can further complicate this distinction because chronic inflammatory skin disease may produce reactive dermatopathic lymphadenopathy with marked nodal enlargement that clinically and radiographically resembles lymphoma. Consequently, prominent lymphadenopathy in a patient with erythroderma may require tissue diagnosis to distinguish a reactive inflammatory process from an underlying lymphoproliferative disorder [4,5].

## Case presentation

A 25-year-old African-American man with chronic psoriasis and asthma presented with progressive bilateral lower-extremity edema and diffuse cutaneous changes. Over the preceding six months, the patient had multiple local emergency department visits for increasing edema and worsening skin disease. He reported that his plaque psoriasis had slowly progressed into near-generalized erythroderma, involving greater than 80% of his body surface area. He also reported a total of 30-lb weight gain over six months, with marked anasarca. Two months prior to admission, his legs were so swollen that he could no longer wear his jeans comfortably (Figure 1, panel A). Despite increasing his exercise in an effort to lose weight, the patient developed worsening edema and difficulty with minimal exertion. Previous courses of oral corticosteroids with tapering and furosemide provided only transient benefit followed by relapse, which significantly frustrated the patient.

**Figure 1:**
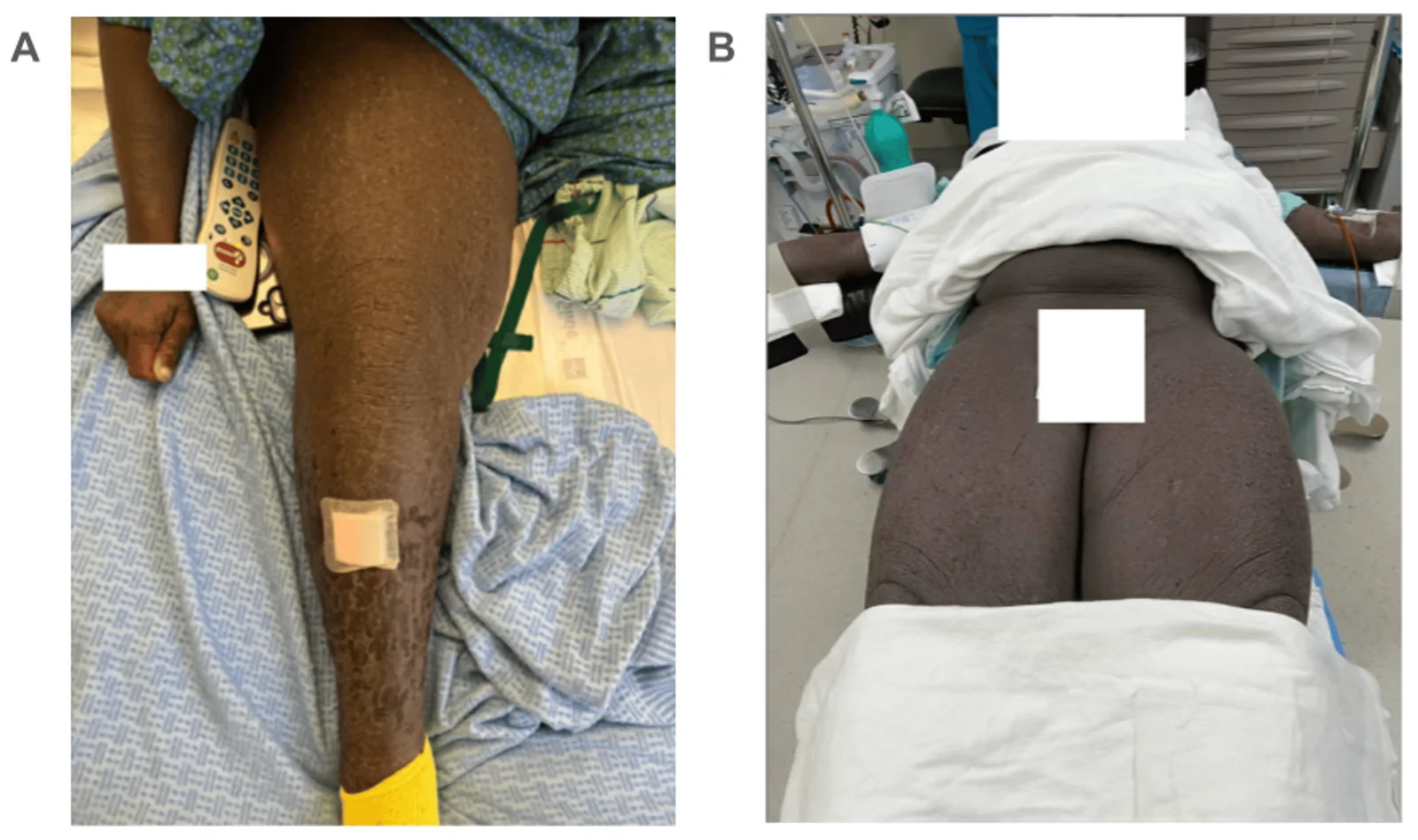
Cutaneous findings before admission and at admission. (A) Two months before admission, scaling and thickening of the skin over the lower extremity were observed. (B) At admission, extensive cutaneous involvement and swelling were observed. The extent of erythroderma and pitting edema was assessed clinically.

On admission, the patient was tachycardic (heart rate: 117 beats/min) but otherwise hemodynamically stable. Physical examination was notable for diffuse exfoliative dermatitis with lichenification, marked 3+ pitting edema of both lower extremities extending to the thighs, and palpable bilateral axillary and inguinal lymphadenopathy. The cutaneous findings are shown in Figure 1, panel B. C-reactive protein was elevated at 2.5 mg/dL. Renal function was preserved with a creatinine of 0.96 mg/dL and an estimated glomerular filtration rate of 112 mL/min/1.73 m^2^; subsequent urine protein studies showed no abnormal proteinuria. Other pertinent laboratory findings are summarized in Table 1.

**Table 1:** Pertinent laboratory findings on admission.

Laboratory test	Patient value	Reference interval
Hemoglobin	11.7 g/dL	13.5-17.5 g/dL
Erythrocyte sedimentation rate (ESR)	11 mm/h	<15 mm/h
C-reactive protein (CRP)	2.5 mg/dL	<0.8 mg/dL
Interleukin-6 (IL-6)	4 pg/mL	<5.0 pg/mL
Lactate dehydrogenase (LDH)	716 U/L	140-280 U/L
Uric acid	9.6 mg/dL	3.4-7.0 mg/dL
Serum albumin	2.8 g/dL	3.1-4.5 g/dL
Total protein	5.4 g/dL	6.2-8.0 g/dL
Serum creatinine	0.96 mg/dL	0.7-1.5 mg/dL
Estimated glomerular filtration rate (eGFR)	112 mL/min/1.73 m^2^	>59 mL/min/1.73 m^2^
Thyroid-stimulating hormone (TSH)	0.036 mIU/L	0.4-4.7 mIU/L
Prolactin	17.8 ng/mL	2.8-15.6 ng/mL
Total testosterone	103 ng/dL	220-780 ng/dL

CT imaging of the chest, abdomen, and pelvis showed hepatomegaly, diffuse anasarca, and bulky bilateral axillary and inguinal lymphadenopathy. Axillary lymph nodes measured up to 3.4 cm on the right and 2.0 cm on the left, with no obvious mediastinal or hilar adenopathy. Representative axillary and inguinal lymphadenopathy is shown in Figure 2, panels A and B. Transthoracic echocardiography showed elevated left ventricular filling pressures, mild pulmonary hypertension, and right ventricular dilation with preserved ejection fraction. These findings alone did not establish a high-output state.

**Figure 2:**
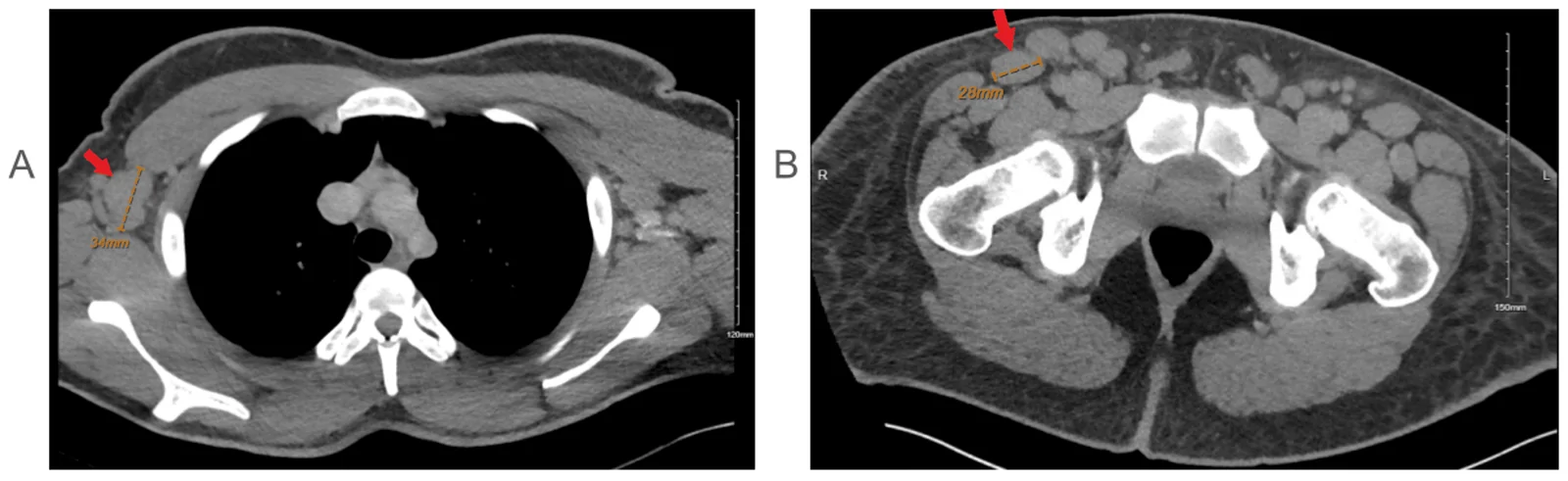
Axillary and inguinal lymphadenopathy on computed tomography. (A) Axial chest CT demonstrating an enlarged right axillary lymph node measuring approximately 34 mm. (B) Axial pelvic CT demonstrating an enlarged right inguinal lymph node measuring approximately 28 mm. Red arrows indicate the enlarged lymph nodes.

Excisional lymph-node biopsy showed dermatopathic lymphadenopathy without evidence of malignancy in the sampled node. Skin biopsy initially showed psoriasiform dermatitis; however, due to persistent concern for lymphoma, specimens were submitted to our regional tertiary academic center for further expert review. This review identified a mixed inflammatory infiltrate with reactive CD30-positive T cells, favoring an inflammatory process over cutaneous T-cell lymphoma.

With pathology favoring an inflammatory process, oral methotrexate (15 mg once weekly) was initiated with concurrent folic acid. Following treatment, the patient reported marked improvement in edema and skin changes. He also received furosemide 20 mg daily for two weeks. At telephone follow-up at three and six months, he reported a return to his functional baseline and a 25-lb weight loss. His recovery was marked by his ability to comfortably wear his jeans again (Figure 3).

**Figure 3:**
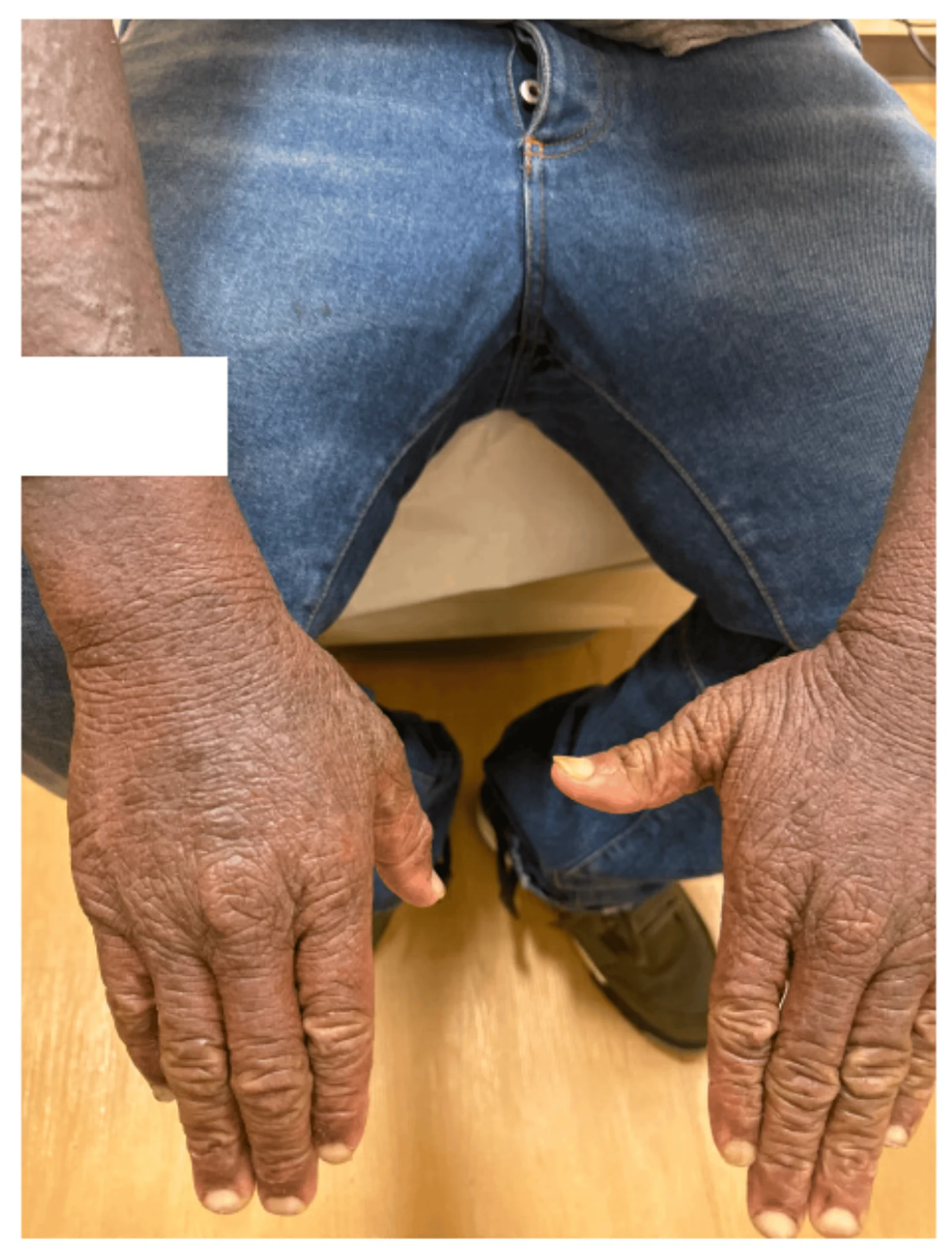
Patient-reported functional improvement. Photograph at three-month follow-up showing the patient wearing jeans again, consistent with his reported improvement in clothing tolerance. Lower-extremity edema cannot be assessed from this photograph.

## Discussion

This case shows that severe erythrodermic psoriasis can be accompanied by substantial systemic illness and marked lymphadenopathy that may closely resemble lymphoma. Diffuse cutaneous vasodilation in erythroderma can reduce systemic vascular resistance and increase cardiac workload, and high-output heart failure is a recognized complication [1,2]. A recent systematic review also noted that erythrodermic psoriasis may present with tachycardia, dehydration, electrolyte disturbances, and lymphadenopathy, reflecting the extent of systemic involvement that can occur [6]. In this patient, the echocardiogram did not establish high-output heart failure. However, his tachycardia, extensive erythroderma, anasarca, hypoalbuminemia, and abnormal echocardiographic findings were consistent with severe systemic disease with cardiovascular involvement. His 30-lb weight gain and anasarca were likely multifactorial, with contributions from cutaneous inflammation, hypoalbuminemia, altered vascular permeability, and fluid retention.

The laboratory findings were also notable. Despite extensive skin disease, the ESR was normal, and serum interleukin-6 was within the reference range, while CRP was elevated at 2.5 mg/dL. Conventional inflammatory markers therefore did not coincide with the clinical severity of his erythroderma. Elevated LDH and uric acid were compatible with increased metabolic and cellular turnover, although neither finding is specific for psoriasis. The thyroid, prolactin, and testosterone abnormalities were also nonspecific and did not establish a discrete endocrine mechanism for his presentation. Erythroderma itself is a clinical syndrome rather than a single diagnosis. In a prospective study of 309 patients with erythroderma, psoriasis accounted for 16.8% of cases, Sézary syndrome for 12.3%, and mycosis fungoides for 5.5% [7]. Therefore, laboratory abnormalities must be interpreted in the context of clinical findings and pathology.

The major diagnostic challenge in this case was distinguishing severe inflammatory erythroderma from lymphoma. This distinction can remain difficult even after an initial skin biopsy. Guglielmo et al. described patients referred with presumed erythrodermic psoriasis who were ultimately found to have lymphoma, demonstrating the degree of clinical overlap between these disorders [3]. Histopathology may also be nondiagnostic in erythroderma. In one retrospective series, blinded histopathologic review correctly identified the underlying diagnosis in only 61% of cases [8]. Reviews of erythrodermic cutaneous T-cell lymphoma recommend correlation of skin histology with peripheral blood studies, immunophenotyping, molecular testing, and lymph-node evaluation when clinical suspicion remains [9]. In our patient, the initial skin biopsy showed psoriasiform dermatitis, while expert review later identified reactive CD30-positive T cells favoring an inflammatory process. The concordant skin and excisional lymph-node findings favored a reactive inflammatory process over lymphoma.

Dermatopathic lymphadenopathy was an important component of the lymphoma-like presentation. It is a reactive form of paracortical lymph-node hyperplasia associated with chronic inflammatory skin disease and commonly involves axillary and inguinal nodes, matching the distribution seen in our patient [4]. In patients with severe dermatologic disease, lymph-node enlargement and metabolic activity can be striking enough to mimic lymphoma on imaging. A similar presentation has been reported in a patient with psoriasis and dermatopathic lymphadenitis who developed extensive fluorodeoxyglucose-avid lymphadenopathy involving multiple nodal stations [5]. In our patient, the bulky axillary and inguinal lymphadenopathy likewise required tissue diagnosis rather than attribution to psoriasis alone.

The subsequent clinical course was also consistent with severe inflammatory skin disease as a major contributor to his systemic illness. Methotrexate remains an established systemic treatment for erythrodermic psoriasis and is among the most frequently reported conventional systemic therapies in the literature [6]. After treatment, the patient reported marked improvement in his skin disease, edema, and functional status, with a 25-lb weight loss and return to baseline activity. Low-dose methotrexate is also active in mycosis fungoides and Sézary syndrome, and the concurrent short course of furosemide may have contributed to improvement in edema [10]. Follow-up was primarily based on telephone interviews, limiting objective assessment of skin clearance and edema resolution. Nevertheless, the clinical course, together with concordant skin and lymph-node pathology, favored severe erythrodermic psoriasis with dermatopathic lymphadenopathy as the unifying diagnosis.

## Conclusions

Severe inflammatory skin disease can cause systemic illness that closely resembles malignancy. The skin should remain part of the differential when evaluating otherwise unexplained systemic findings, particularly when lymphadenopathy is present. In patients with erythroderma and marked adenopathy, reactive dermatopathic lymphadenopathy and lymphoproliferative disorders should both be considered, with diagnosis guided by correlation of the clinical presentation with skin and lymph-node pathology. Recognizing this overlap early may reduce diagnostic anchoring on malignancy while still ensuring that lymphoma is appropriately excluded. In complex cases, response to treatment can provide additional clinical information, but it should not replace diagnostic evaluation or be used alone to exclude lymphoma.
